# Atypical Focal Osteomyelitis as Initial Manifestation of AIDS

**DOI:** 10.1155/2011/541873

**Published:** 2011-09-25

**Authors:** A. Akiki, Y. Bilde

**Affiliations:** Traumatology Unit, Chablais Hospital, 1870 Monthey, Switzerland

## Abstract

Persistent pain development after a skeletal contusion rarely poses the diagnosis of osteomyelitis. We report the case of a fibular head contusion as an initial manifestation of a focal abscess development in a healthy young patient. The traditional treatment of surgical drainage revealed the presence of an atypical *Mycobacterium haemophilum* isolates in the abscess. This lead to further investigations that concluded and established the diagnosis of AIDS. *Conclusion*. Isolation of an atypical *Mycobacterium haemophilum* in any abscess should lead the physician to accomplish further investigations and look for AIDS even in young healthy subjects.

## 1. Case Report

A 24-year-old, Vietnamese male, living in Switzerland for the last three years, consulted our unit for unremitting pain in his right knee. He had a right knee contusion two months ago. On history, he related occasional night sweats with chills since that accident accompanied with a weight loss of 5 kg. Otherwise, he is in good health, does not smoke, and takes no medications.

**Figure 1:**
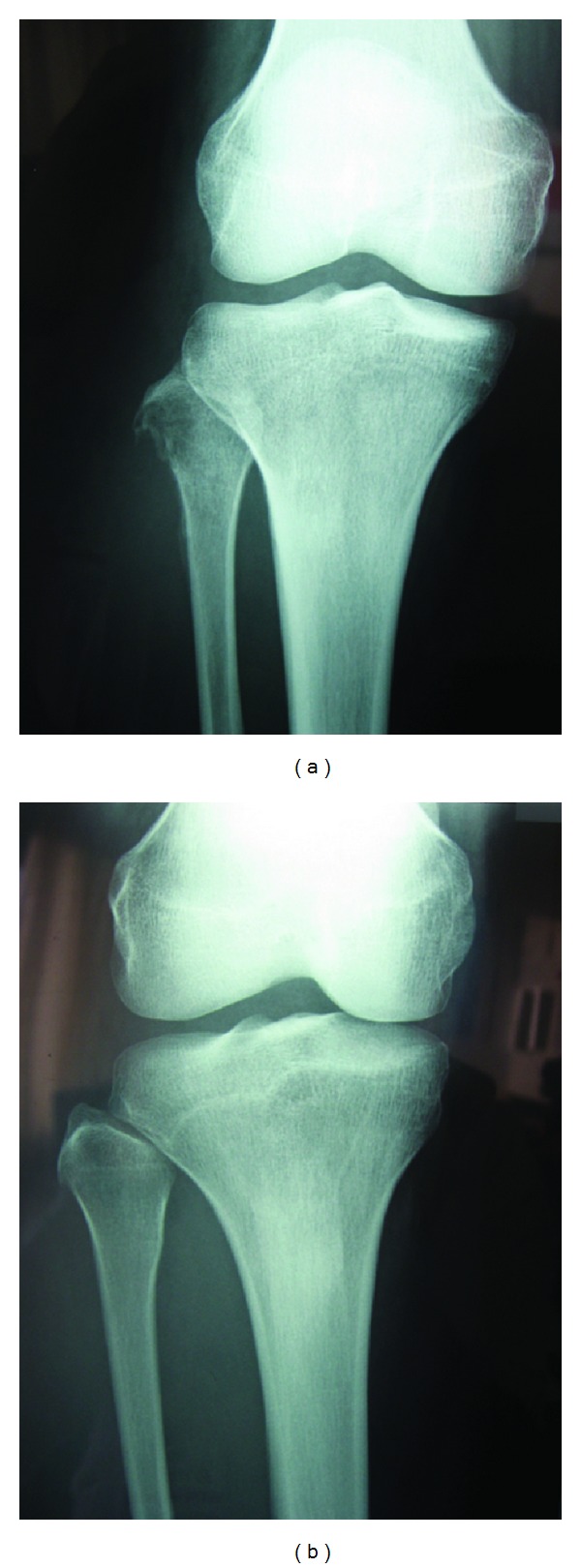
(a) 2 months later, the lytic lesion has rapidly progressed and destroyed the external cortical bone of the proximal peroneal head. (b) Initial right knee radiography where we notice a small bony spicule at the proximal fibula head.

**Figure 2:**
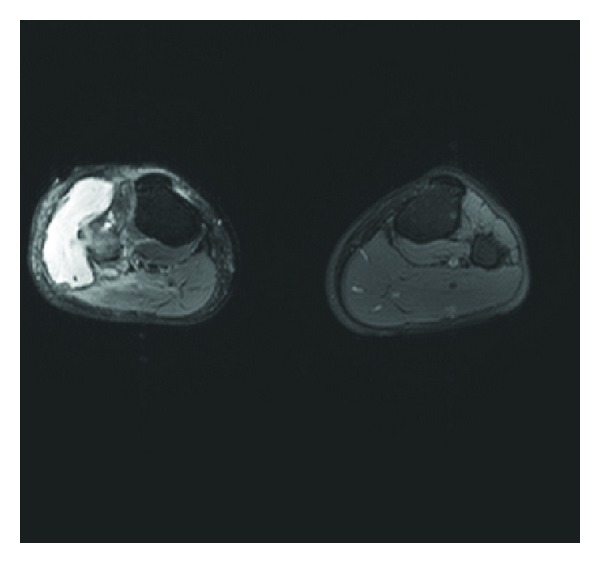
Sagittal MRI section of the lower limbs. We notice the presence of a T2 hypersignal at the level of the proximal right fibular head, with erosion of the lateral cortical bone and extension into adjacent soft tissues in the form of a collection.

Clinical examination showed a painful red nonulcerating indurations measuring 3 × 4 cm in surface at the level of the right fibular head. It is fluctuating and seems to be adherent to the deep subcutaneous plane. Right knee mobilisation was complete, and neurovascular examination was normal. Blood analysis revealed a marked microcytic anaemia (haemoglobin: 76 g/L, VGM: 61 fl), no leukocytosis, and a CRP of 4 mg/L. Knee radiography showed a lytic lesion with ill-defined margin in the proximal right fibula measuring 1 × 1 cm and breaking through the outer cortex (Figure 1(a)). We reserved the differential diagnosis of focal osteomyelitis versus a tumoral process since the radiography showed that progressive lesion (Figure 1(b)). MRI examination demonstrated a hypersignal on T2 images of the proximal right fibular head with breakage through the outer cortical bone and an abscess extension in the surrounding soft tissues. This excluded the tumoral process as a causal agent and oriented us to a focal osteomyelitis with local infiltration of peripheral tissues (Figure 2). Surgical debridement was done through a centred lateral incision and tissue fragments sent for bacteriology and path anatomy analysis with a special request to look for tuberculous infection. This atypical request was based on the ethnical origin of the patient and the lytic lesion on radiography. Antibiotic therapy has been started empirically with Augmentin (amoxicillin and clavulanic acid) at a dose of 2, 2 g intravenous three times a day. The path anatomical analysis revealed the presence of an acid-alcohol resistant bacillus within an inflammatory process. Bacteriological results were noncontributive. A mycobacterium infection as the source of the osteomyelitis is suggested, but the IDR (intradermal reaction to tuberculin) tuberculosis test came out negative after 72 hours.

Locally, scar adhesion developed, a second surgical debridement was done, and new bone and tissue fragments were sent for bacteriology and molecular biology for analysis in order to identify the type of mycobacterium.

In the meanwhile, we completed and widened our investigations looking for any signs of immunodepression. Our results confirmed the presence of an HIV infection diagnosed as AIDS with a CD4 count less than 65/mm^3^ and a CD4/CD8 ratio of 0.035. It also revealed the presence of a viral hepatitis C infection. An antipneumocystic prophylaxis with Bactrim Forte (1 pill three times a week) was started. PCR (polymerase chain reaction) results of the last fragments analysed show the presence of an atypical nontuberculous mycobacterium identified as *Mycobacterium haemophilum*. Antimycobacterial treatment with Clarithromycin (500 mg/day), Moxifloxacine (400 mg/day), and Rifabutin (300 mg/day) was started for a minimum of 12 months. Secondarily, an antiretroviral treatment was started 2 weeks later. Locally, the patient is doing now well and is treated by our infectious disease department team for further followup and control.

## 2. Discussion

*Mycobacterium haemophilum* is a nontuberculose atypical mycobacteria with a slow evolution and a frustrating clinical presentation [1]. It was described first in 1978 by Sompolinsky et al. in a young patient presenting chronic cutaneous ulcerations with the background of Hodgkin disease [2]. Till year 2000, only 90 cases of *Mycobacterium haemophilum* infections were reported across the world [3]. Clinical symptoms essentially manifest as chronic nodular or ulcerative skin lesions, but rare cases of bacteraemia, pneumopathies, septic arthritis, and osteomyelitis have been reported [1, 4, 5].

Ways of transmission are still unknown, but, in the majority of cases, the cellular immunity status of the patient seems to be a determinant factor in disease manifestation. All patients who manifested this infection had in common a depressed immunostatus state [1].

It is in 1987 that Males et al. [6] reported for the first time a *Mycobacterium haemophilum* infection in an HIV patient. During the AIDS epidemic, the incidence of *Mycobacterium haemophilum* infection has increased to a point that its implication should be considered in every differential diagnosis of an osteolytic lesion in any AIDS patient. In a three-year prospective study including 25 patients infected with the HIV virus and presenting a focal osteomyelitis due to atypical mycobacteria, *Mycobacterium haemophilum* was imputed in 44% of cases, followed by Hirsch et al. [7]. *Mycobacterium haemophilum* is thus the most frequent pathogen causing osteomyelitis in AIDS patients [7–9]. More than 30 cases were reported with a majority of patients with advanced AIDS disease or chronically immunosuppressed [1, 4, 10–12].

The bony lesion can be focally limited or can spread to the skeleton. Contamination is thus contiguous or haematogenous [13].

As most of mycobacterial infections, clinical symptoms develop slowly with a tendency to chronicity. This microorganism is difficult to cultivate in standard milieu of bacteriology. Its identification requires specific milieu at a temperature varying between 30–32° Celsius [1]. With the new improvement in molecular biology, PCR examination or the 16S ribosomal RNA gene sequencing its identification however became easier [14–16].

*Mycobacterium haemophilum* is resistant to classic antituberculous drugs as INH, Pyrazinamide, and Ethambutol. In 56% of cases, it does not respond to Rifampicin [9]. The first intention treatment is thus a tritherapy combination of Ciprofloxacin, Clarithromycin, and Rifabutin for 9–18 months. In cases of irreversible immunosuppression, patients should continue this tritherapy for life [1, 3, 4, 10, 17–19].

Parallel surgical debridement of the infected site seems to favour the efficiency of the medical treatment and should be done as possible [20].

In all cases, reversing the immunosuppression status of the patient, when this is possible, seems to be the key success for the treatment giving the very low pathogenicity of *M. haemophilum* in immunocompetent patients [21].

## 3. Conclusion

*Mycobacterium haemophilum* is significantly pathogenic in immunocompromised patients. If an apparent healthy patient presents with a focal osteomyelitis with an atypical mycobacterium, a search for an immunosuppressed status should be looked for. AIDS should be considered in the differential diagnosis of an apparent healthy patient with a *Mycobacterium haemophilum* infection, since this infection is almost pathognomonic for immunosuppressed status. The diagnosis will be evoked essentially in patients with high index of suspicion and when the bacterial examination reveals the presence of BAAR. The success of treatment relies on long-term tritherapy combined with surgical debridement of the infected site and the reversal of the immunosuppression if possible.
